# Olanzapine-Induced Acute Pulmonary Embolism

**DOI:** 10.7759/cureus.68626

**Published:** 2024-09-04

**Authors:** Jihane Moussaoui, Ikram Saadi, Mohammed Barrimi

**Affiliations:** 1 Psychiatry, Faculty of Medicine and Pharmacy, Mohammed I University, Oujda, MAR

**Keywords:** anti psychotic agent, chest pain, antipsychotics, olanzapin, pulmonary embolism

## Abstract

Pulmonary embolism is defined as the abrupt obliteration of the trunk or a branch of the pulmonary artery by an embolus most often from a deep vein thrombosis of the lower limbs. It is serious, underdiagnosed, and can be life-threatening. We report the case of a patient who presented with a massive acute pulmonary embolism while taking olanzapine. The interest of our case lies in its rarity, its seriousness but also the possibility of prevention and adequate management in the case of any suggestive clinical symptoms.

## Introduction

Pulmonary embolism is defined as the abrupt obliteration of the trunk or a branch of the pulmonary artery by an embolus most often from a deep vein thrombosis of the lower limbs. It is serious, underdiagnosed, and can be life-threatening [1].

The risk factors for pulmonary embolism can be classified into two groups:

- Hereditary factors such as genetic mutations affecting factor V of Leiden and prothrombin or factor II and the congenital deficiency of physiological inhibitors of coagulation such as antithrombin and protein C and S [1].

- Acquired factors such as recent surgical interventions (particularly orthopedic surgery, oncological surgery, and neurosurgery), trauma, immobilization or prolonged bed rest for more than five days, hormone replacement therapy and contraceptive pills, active neoplasms, obesity, smoking, history of thromboembolism, hospitalization, infection, pregnancy, and postpartum [2,3].

Other disorders have been associated with the high risk of pulmonary embolism including heart failure, ischemic stroke, acute respiratory failure or intubation, sepsis, and inflammatory bowel disease [4]. Pulmonary embolism is a multicausal pathology; however, it can occur in patients with no identified risk factor. The risk of thromboembolism is higher in psychiatric patients compared to the general population, with an incidence of up to 11.6% at three months of treatment [3].

We report the case of a patient who presented with a massive pulmonary embolism while taking olanzapine. The interest of our case lies in its rarity, its seriousness but also in the possibility of prevention and adequate management in the face of any suggestive clinical symptoms.

## Case presentation

We report the case of a 55-year-old male patient without thromboembolic risk factors, and having cardiovascular risk factors as age, male gender, overweight (body mass index at 26.5 kg/m²), and psychiatric history; several cousins followed for chronic psychoses and mood disorders, one of which committed suicide.

The patient was hospitalized in the psychiatric department for the management of psychomotor agitation. On admission he was conscious, calm on the motor level, and well-oriented, his outfit was clean and adapted, his concentration and attention were normal, and his contact was superficial. He was hypomimic and his mood was sad. The course of thought was continuous, and the content was marked by the presence of a delirium of persecution toward those around him. He also reported auditory hallucinations with disturbed judgment and negative insight.

Based on clinical and anamnestic data, the diagnosis of an acute psychotic episode was made and the patient was placed on olanzapine 10 mg/day and chlorpromazine 75 mg/day.

The evolution was marked by the onset of mental confusion on the second day of treatment, dyspnea with palpitations, and disturbance of consciousness. The patient was hypertensive at 160/110 mmHg, tachycardic at 115 beats per minute, and polypneic with a respiratory rate of 25 cycles per minute, had an oxygen saturation at 97% in ambient air and blood sugar at 1.04 g/l without the notion of chest pain or syncope, and he was transferred to the unit of intensive cardiological care.

He had an ECG that showed sinus tachycardia, a chest X-ray that showed an ascension of the right diaphragmatic dome, a complete biological workup with a normal blood count, a correct renal and hepatic function, a correct ionogram, a normal hemostasis, CRP (C-reactive protein) at 152 mg/l, CPK (creatine phosphokinase) at 453 IU/l, ultrasensitive troponin positive at 82 ug/l, positive D-dimers, proBNP (pro-brain natriuretic peptide) at 4569 pg/ml, and a negative lumbar puncture.

**Figure 1:**
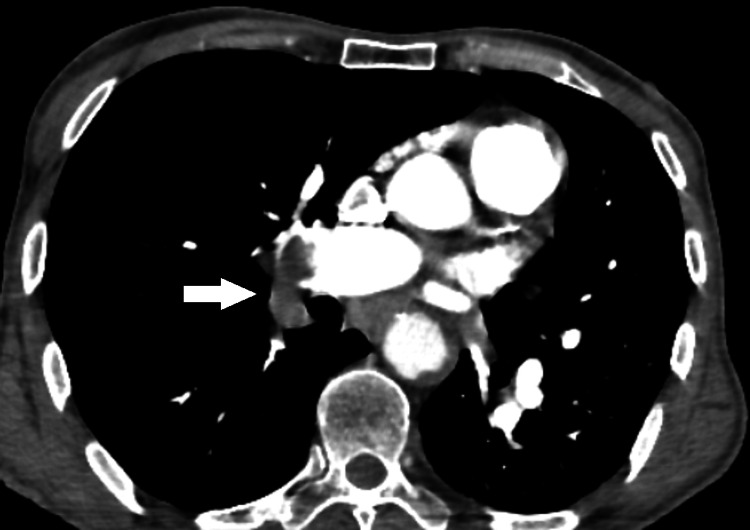
CT pulmonary angiography This axial section, in arterial time, shows hyperdense material within the right pulmonary artery extending to its lobar branches corresponding to the thrombus.

A cerebral CT scan with an injection of contrast product returned without peculiarities; echocardiography showed an undilated left ventricle, hypertrophied and of good systolic function, 60% ejection fraction, a dilated left atrium, a dilated right ventricle of systolic function borderline with a dry pericardium, and an inferior vena cava dilated and not very compliant. A CT pulmonary angiography revealed a bilateral pulmonary embolism as shown in Figure 1.

The diagnosis of a pulmonary embolism at high intermediate risk (positive troponin, high proBNP, and a dilated right ventricle of systolic function borderline) was made. The patient received rivaroxaban 30 mg/day and compression stockings were indicated. As part of the etiological assessment, we performed a cerebral-cervico-thoraco-abdomino-pelvic CT scan which returned without abnormalities.

In addition, we did not objectify a personal antecedent of malignancy, nor any notion of bed rest or physical restraint during hospitalization or a family history of thrombophilia. During his follow-up and after multiple psychiatric interviews with the patient and his family, the diagnosis of a major depressive episode was retained.

The management was to stop olanzapine and chlorpromazine and the patient was put on paroxetine 20 mg/day with regular follow-up in consultation and good improvement. His anticoagulant treatment was maintained for three months and then stopped after a gradual decline without any recurrence until 12 months of follow-up.

## Discussion

The clinical presentation of pulmonary embolism is variable and sometimes atypical depending on the degree of involvement and the underlying field. It can appear as a dyspnea with chest pain progressively worsening as it can lead suddenly to death.

With a massive pulmonary embolism, the clinical presentation is louder with threatening cardiac dysfunction and hemodynamic and respiratory instability. In fact, it is recommended to use standardized algorithms to assess the clinical probability and clearly indicate the biological and radiological investigations and therefore avoid any unnecessary exploration [5,6]. For example, Borkowski et al propose a stratification based on the assessment of cardiac biomarkers, namely BNP, NT-proBNP, and troponin complex [7].

By referring to anamnestic, clinical, and paraclinical data, we strongly incriminate antipsychotics and more particularly olanzapine more than chlorpromazine in the occurrence of pulmonary embolism in our patient. Our arguments in favor of the accountability of olanzapine are as follows:

 - The introduction of olanzapine is recent and the patient has never been put on this drug.

 - Our patient was put on chlorpromazine for his insomnia for two years without any thromboembolic events.

- According to the data in the literature, olanzapine seems more implicated than chlorpromazine in the occurrence of pulmonary embolism.

Several studies have shown that antipsychotics such as olanzapine, clozapine, and quetiapine constitute independent risk factors for thromboembolic events, particularly at the initiation of treatment [8,9]. A meta-analysis comparing 31,095 cases of pulmonary embolism with more than 143,000 control cases indicates that the incidence of thromboembolic events is 139% higher in patients on antipsychotics [10].

Studies report that women treated with risperidone, chlorpromazine, levopromazine, and haloperidol are more exposed than men. Like the case of our patient, Toringhibel et al. reported the case of a patient treated with olanzapine and in whom the diagnosis of massive pulmonary embolism was made and he was treated with thrombolytics [11].

Allenet et al. have also reported an increased risk with olanzapine, clozapine, and risperidone [12]. In the elderly, the antipsychotics involved in particular are olanzapine, clozapine, risperidone, and quetiapine [13]. Clozapine is associated with the highest risk of this disease.

According to data from the World Health Organization, thromboembolic events have been reported more frequently with olanzapine, sertindole, and zuclopenthixol compared to other neuroleptics [14].

Exposure to antipsychotics increases the risk of pulmonary embolism regardless of age, sex, duration of anticoagulation if a history of pulmonary embolism, and the initial clinical picture. The acute decompensation of a chronic psychiatric disorder is also a risk factor [15]. It has also been suggested that the risk of thromboembolism is much more linked to the underlying psychiatric disorder than to the treatments.

Publications have shown that certain psychiatric pathologies are particularly at greater risk for thromboembolism by being themselves thrombogenic. Strudsholm et al. have reported that patients with bipolar disorder presented significantly recurrent pulmonary embolism and two studies have shown an association between depression and venous thromboembolic disease [16,17]. Thus, our patient associates two probable risk factors: depression and antipsychotics.

A state of hypercoagulability and hypofibrinolysis has been found in schizophrenic patients and those presenting an acute psychotic episode exposing them more frequently to these accidents [18]. A relative added risk of thromboembolic disease and pulmonary embolism has been reported in recurrent depression, bipolar disorder, and schizophrenia, compared to the general population [19,20].

The risk is increased with polytherapy and depends on the type and dose administered, particularly when an atypical antipsychotic is combined with a typical antipsychotic. One study reports that the case of a young male patient treated with olanzapine and risperidone presented three successive embolic episodes, and his only risk factor was smoking [19-21].

Although the exact pathophysiological mechanism by which antipsychotics induce pulmonary embolism is not entirely well understood, several hypotheses have been put forward. For example, venous stasis and sedentary lifestyle caused by disease or sedation induced by antipsychotics, iatrogenic weight gain, the increase induced by chlorpromazine, the presence of anti-phospholipid antibodies, platelet aggregation activated by the serotoninergic system, hyperleptinemia as well as metabolic complications [21].

The prothrombotic side effects of certain antipsychotics may also be involved and then patients receiving conventional antipsychotics seem to have higher concentrations of anti-phospholipid antibodies, including anticoagulants and anti-cardiolipin antibodies. The presence of these antibodies is associated with a high risk of thromboembolism. It also appears that mental illness is accompanied by an increase in pro-coagulant activity, and patients with acute psychosis have shown a statistically significant increase in the level of D-dimers, P-selectin, and receptor expression glycoproteins IIb/IIIa on the surface of platelets.

The metabolites of olanzapine can induce thrombosis by causing arterial hypotension, venous stagnation in the extremities, and thrombus formation. The risk of embolic recurrence with antipsychotics and after reduction of anticoagulation remains unknown, and taking antipsychotics after stopping anticoagulation may again increase the risk of thromboembolism. Thus, the pharmacological management of a psychiatric patient after an embolic episode is a real challenge.

There are no guidelines concerning the management of pulmonary embolism in patients treated with antipsychotics, but anticoagulation prolonged over at least three months is recommended in all patients with pulmonary embolism. All the probable thromboembolic risk factors must be taken into account in the pharmacological choice before the administration of antipsychotics in psychiatric patients.

Other preventive measures such as rehydration, compression stockings, regular mobilization, particularly of lower extremities, and preventive anticoagulation with low doses of low-molecular-weight heparin may also be offered if the case is favorable [20,21].

## Conclusions

Pulmonary embolism is a predictable fatal accident but very often underdiagnosed, thus reducing the chances of survival, so once suspected, a rapid and targeted diagnostic and therapeutic approach must be implemented taking into consideration the risk-benefit ratio for the patient. The diagnosis and prevention of pulmonary embolism are the major objectives for the reduction of morbidity and mortality in patients treated with olanzapine. Monitoring of the tolerance of psychotropic drugs is mandatory as well as the search for undesirable effects.
